# Development of T Lymphocytes in the Nasal-associated Lymphoid Tissue (NALT) from Growing Wistar Rats

**DOI:** 10.1080/10446670410001670463

**Published:** 2004-03

**Authors:** Gustavo A. Sosa, María E. Roux

**Affiliations:** 1 Laboratory of Cellular Immunology CONICET, Faculty of Pharmacy and Biochemistry Department of Biological Sciences University of Buenos Aires. Junín 956 5th floor Buenos Aires C1113AAD Argentina

## Abstract

The aim of the present report was to study the development of several T-lymphocyte subsets in the nasal-associated lymphoid tissue (NALT) of growing Wistar rats. CD5+ and CD4+ lymphocytes gradually increased with age. A predominance of CD8α+ over CD4+ T cells was found from 7 to 45 days but from 45 to 60 days of age T helper cells outnumbered the cytotoxic subpopulation. The majority of CD8+ T lymphocytes expressed the heterodimeric isoform. The most relevant findings by immunohistochemistry are: (1) the predominance of TCRγδ+ and CD8α+ cells at 7 days postpartum over all the other T-cell subpopulations; and (2) that TCRγβ+ outnumbered TCRαβ+ T cells from 7 to 45 days postpartum whereas αβ T cells predominated in 45- and 60-day-old rats. Besides, cytometric studies have shown that the percentages of TCRγ+, CD8+, as well as the population coexpressing both phenotypes (TCRγδ+CD8α+), were significantly higher in rats at 7 days postpartum when compared to 60 day-old rats. In the present study, the finding of a high number of γδ+ and CD8+ T cells early in NALT development may indicate the importance of these subpopulations in the protection of the nasal mucosa in suckling and weaning Wistar rats.


*Clinical & Developmental Immunology*, March 2004, Vol. 11 (1), pp. 29–34

Taylor & Francis healthsciences

### Development of T Lymphocytes in the Nasal-associated Lymphoid Tissue (NALT) from Growing Wistar Rats

GUSTAVO A. SOSA and MARÍA E. ROUX*

*Laboratory of Cellular Immunology, CONICET, Faculty of Pharmacy and Biochemistry, Department of Biological Sciences, University of Buenos Aires, Junín 956 5th floor, C1113AAD, Buenos Aires, Argentina*

The aim of the present report was to study the development of several T-lymphocyte subsets in the nasal-associated lymphoid tissue (NALT) of growing Wistar rats. CD5+ and CD4+ lymphocytes gradually increased with age. A predominance of CD8α+ over CD4+ T cells was found from 7 to 45 days but from 45 to 60 days of age T helper cells outnumbered the cytotoxic subpopulation. The majority of CD8+ T lymphocytes expressed the heterodimeric isoform. The most relevant findings by immunohistochemistry are: (1) the predominance of TCRγδ+ and CD8α+ cells at 7 days postpartum over all the other T-cell subpopulations; and (2) that TCRγδ+ outnumbered TCRαβ+ T cells from 7 to 45 days postpartum whereas αβ T cells predominated in 45- and 60-day-old rats. Besides, cytometric studies have shown that the percentages of TCRγδ+, CD8α+, as well as the population coexpressing both phenotypes (TCRγδ+CD8α+), were significantly higher in rats at 7 days postpartum when compared to 60 day-old rats. In the present study, the finding of a high number of γδ+ and CD8+ T cells early in NALT development may indicate the importance of these subpopulations in the protection of the nasal mucosa in suckling and weaning Wistar rats.

*Keywords*: CD8+ T cells; Development; Growing Wistar rats; NALT; TCRγδ+ T cells

### INTRODUCTION

The mucosal membranes of vertebrates are weak mechanical barriers that possess extensive innate and specific mechanisms of defence. The innate mechanisms have been extensively reviewed elsewhere (Pruitt *et al.*, 1999; Diamond *et al.*, 2000).

A morphologically and functionally distinct line of protection characteristic of mucosal surfaces is the so-called mucosa-associated lymphoid tissue (MALT). MALT refers to solitary lymphoid nodules and large organised collections of lymphoid tissues located in the lamina propria of the mucosal membranes. The tissues that are part of the MALT include the middle ear, parts of the urogenital tract, the mammary gland, the conjunctiva, the salivary glands, the nasopharyngeal lymphoid tissue (NALT), as well as bronchus-associated lymphoid tissue (BALT) and gut-associated lymphoid tissue (GALT) (Croitoru and Bienenstock, 1994).

MALT is characterised by the predominance of local dimeric IgA production, secreted as secretory IgA (sIgA) that is responsible for the immune exclusion of bacteria and viruses (Mazanec *et al.*, 1993). The term common mucosal immune system (CMIS) implies that activated lymphocytes derived from one mucosal surface can recirculate and localise selectively in other mucosal surfaces. This connection between different mucosal surfaces permits immunity initiated at one anatomical site to protect other mucosal sites (McDermott and Bienenstock, 1979).

In the upper respiratory tract, MALT is represented by nasal-associated lymphoid tissue (NALT) and the term was coined by different authors (Mair *et al.*, 1987; Spit *et al.*, 1989). In humans, NALT is known as Waldeyer's ring and it consists of the adenoids or nasopharyngeal tonsil, the bilateral pharyngeal lymphoid bands, the bilateral tubal tonsils around the opening of the two Eustachian tubes, the bilateral palatine tonsils at the lateral opening of the oropharynx, and the lingual tonsil.

Tissues equivalent to Waldeyer's ring have been found in cattle, monkeys (Loo and Chin, 1974; Harkema *et al.*, 1987), horses (Mair *et al.*, 1987; Mair *et al.*, 1988) and also in chickens (Hodges, 1974). Other authors reported the presence of lymphoid aggregations in mice (Belai *et al.*, 1977; Ichimiya *et al.*, 1991).

Massive lymphoid accumulations have been described in the mucosa of the opposite lateral walls near the anterior orifice of the nasopharyngeal duct when studying the rat upper respiratory tract (Kelemen, 1947). This paired and rod-shaped tissue, situated in the floor of

*Corresponding author. Tel.: +54-11-4801-2864. Fax: +54-11-4508-3645. E-mail: mroux@ffyb.uba.ar

ISSN 1740-2522 print/ISSN 1740-2530 online © 2004 Taylor & Francis Ltd
DOI: 10.1080/10446670410001670463

the nasal cavity, is the only well-organised MALT with a fixed location in the nasopharynx of rodents (Spit *et al.*, 1989; Hameleers *et al.*, 1989; Kuper *et al.*, 1990; van der Ven and Sminia, 1993). Therefore, this tissue can be regarded as the nasopharyngeal tonsil and as an equivalent of Waldeyer's ring because structures similar to the palatine and lingual tonsils have not been observed in rodents.

In rats, NALT is present at birth, earlier than BALT and this is probably due to its strategic position with respect to the incoming air. As rats are obligate nasal breathers, the inspired air, laden with environmental antigens, passes the nasal cavities before it reaches the lungs. Some immunohistochemical studies regarding the ontogeny of lymphoid and non-lymphoid cells have been performed in rats only from birth up to weaning (21 days of age). Furthermore, those concerning the phenotypic characterisation of T lymphocytes were semiquantitative and incomplete due to the lack of some monoclonal antibodies (Hameleers *et al.*, 1989).

Therefore, the aim of the present report was to perform a complete phenotypic study of T-cell subpopulations in the NALT of growing Wistar rats from 7 to 60 days of age.

### MATERIALS AND METHODS

### Animals

Suckling rats 7 and 12 days old, and weaning rats of the Wistar strain (closed colony from the breeding unit kept at the animal facilities of the Faculty of Pharmacy and Biochemistry, University of Buenos Aires, Argentina) of either sex were used. Weaning rats (21 days old) were fed a stock diet (Cargill, Argentina) up to 60 days of age. Water and diet were given *ad libitum*. During all the experimental time animals were subjected to a 12-h light period and room temperature was kept at 21 ± 1°C.

Experiments were performed using 5 or 6 animals per group.

### Isolation of NALT

Rats were killed by decapitation under anaesthesia with diethyl ether at 7, 12, at weaning (21 days), and at 45 and 60 days of age.

The lower jaws and the tongues were removed and the palates were excised with a scalpel blade from behind the incisor teeth to the molar teeth. Then, palates were gently pulled from front to rear with fine forceps, carefully dissecting them from the underlying bone tissue with the scalpel blade.

### Immunohistochemistry

#### *Indirect Immunofluorescence*

Palates, containing NALT on their nasal sides, were immediately placed in 95° ethanol at 4°C in order to be processed by Sainte-Marie's technique (Sainte-Marie, 1962). Briefly, tissues were fixed in 95° ethanol precooled at 4°C, dehydrated in four changes of pre-cooled absolute ethanol, cleared by passing through three consecutive baths of xylene and embedded in paraffin at 56°C. Tissue sections (4–5 μm thick) were placed on glass microscope slides. Paraffin was removed by gently swirling the slides in two consecutive baths of xylene, which was removed in two baths of absolute ethanol, two baths of 95° ethanol and three baths of saline solution (0.9% NaCl w/v in distilled water).

T cells were labelled with the xenogeneic monoclonal antibodies against rat CD5 (clone OX-19), CD4 (clone OX-38), CD8α (clone OX-8), CD8β (clone 341), TCRαβ (clone R73) and TCRγδ (clone V65) (BD PharMingen, San Diego, CA, USA). After an overnight incubation at 4°C, the fluorescein-conjugated goat $F(ab)'_2$ fragment to mouse IgG (whole molecule) (ICN Cappel, Aurora, OH, USA) was added. All tissue incubations were performed in a moist chamber, and sections were washed three times in saline after each incubation step. Control staining was carried out simultaneously omitting the primary antibody step. Tissue sections of NALT were viewed at 1,000 × magnification with an Olympus epifluorescence microscope. Cell counts were performed by the authors in a blind fashion.

#### *Avidin-biotin-peroxidase*

To confirm the presence of certain T-cell phenotypes at 7 days of age we used avidin-biotin-peroxidase-DAB (Vectastain® ABC Kit, Vector Laboratories, Inc, Burlingame, CA, USA) that is the most sensitive immunohistochemical technique.

In brief, tissue sections were deparaffinized and hydrated as explained above. Then the sections were incubated in 0.3% $H_2O_2$ in methanol and, after rinsing in PBS, incubated with diluted horse normal serum. T cells were labelled with the xenogeneic monoclonal antibodies against rat CD8α, CD8β, TCRαβ and TCRγδ. After an overnight incubation at 4°C, the sections were incubated with biotinylated horse anti mouse IgG and then with Vectastain® ABC reagent. Finally, sections were immersed in 1 mg/ml DAB solution in Tris–HCl pH 7.2, rinsed in tap water, counterstained with Harris' haematoxylin, cleared and mounted.

### Preparation of Cell Suspensions

Palates, containing NALT, were immediately placed in a Petri dish containing ice-cold HBSS supplemented with 5% heat-inactivated foetal bovine serum (HBSS/FBS) and teased gently against a stainless steel mesh immersed in the medium in order to release cells. NALT cell suspensions from individual animals were pooled and washed three times with HBSS/FBS by centrifugation. Then, erythrocytes were eliminated by the addition of 0.83% $NH_4Cl$ solution. The dissociated cell suspensions

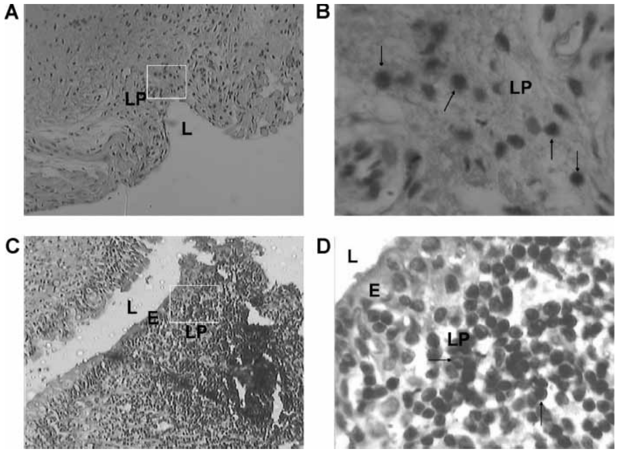

FIGURE 1 Paraffin sections of rat NALT stained with anti TCRγδ monoclonal antibody (avidin-biotin-peroxidase-DAB technique) and counterstained with Harris' haematoxylin. A and B: NALT of a 7-day-old rat. C and D: NALT of a 60-day-old rat. The areas in the white squares from A and C are shown at a higher magnification in B and D, respectively. A and C, 200X; B and D, 1000X. Arrows indicate γδ+ T cells. L: lumen of nasal cavity; E: NALT epithelium; and LP: NALT lamina propria.

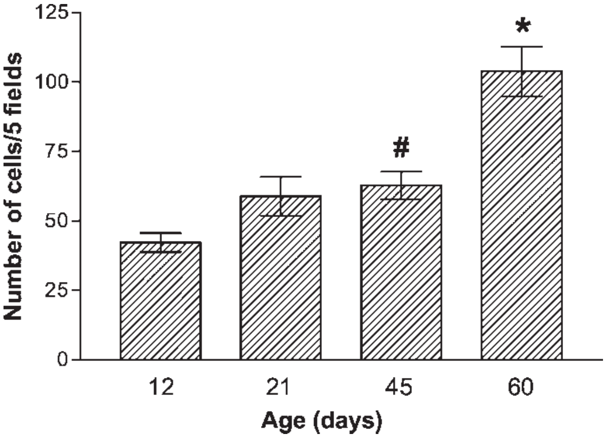

FIGURE 2 Number of CD5+ T cells in NALT from 12-, 21-, 45- and 60-day-old-rats. Bars represent mean ± SE of the number of cells in 5 fields from 5 animals. Tukey-Kramer: (#) 45 vs 60, $p < 0.01$; (*) 12, 21 vs 60, $p < 0.001$.

were filtered through nylon wool to remove large cellular aggregates and detritus and then washed three times with HBSS/FBS by centrifugation. Viability of cell preparations routinely exceeded 90% as judged by Tripan Blue staining.

### Flow Cytometry

Pooled cells were phenotypically characterised using a FACScan flow cytometer (Becton Dickinson, San Jose, CA, USA). Data were collected for 10,000 events. Isotype-matched antibodies were used as negative controls. Staining for T cells was performed using the following monoclonal antibodies (BD PharMingen, San Diego, CA, USA): fluoresceinated (FITC) mouse $IgG_{1,\kappa}$ anti-CD8α (clone OX-8); PE $IgG_{1,\kappa}$ anti-TCRγδ (clone V65).

Results are expressed as the percentage of positively stained cells in the total cell population exceeding the background-staining signal.

### Statistical Analysis

Results are expressed as mean ± standard error(SE) of the absolute number of cells in NALT lamina propria. Due to the very small size of NALT in 7 day-old rats, 2 fields were recorded and 5 fields for 12, 21, 45 and 60 day-old rats.

Statistical evaluation of results was performed with GraphPad InStat version 3.01 (GraphPad Software, San Diego, CA, USA) using 2-tailed Student's *t*-test and one-way ANOVA with Tukey-Kramer's post test, taking $p < 0.05$ as significant.

### RESULTS

At day 7 postpartum NALT consisted of two very small structures in which we observed few lymphocytes (Fig. 1A). All CD5+ T-cell subpopulations were present and there was a predominance of CD8α+ and TCRγδ+ over CD4+ and TCRαβ+ T cells, respectively. Due to the very small size of NALT at this age, data are expressed as number of cells in 2 fields as shown in Table I. TCRγδ+ cells are indicated with arrows in Fig. 1B.

We observed that the absolute number of CD5+ T cells gradually increased with age and that there are only significant differences when we compare the 60-day-old group with 12-, 21- and 45-day-old rats (Fig. 2).

The study performed on CD5+ T-cell subpopulations showed that: (1) CD4+ T cells increased with age but significant differences were only observed between the 45- and 60-day-old rats with respect to the other groups (Fig. 3); and there were no significant differences in the CD8α+ T lymphocytes, with the majority expressing the heterodimeric isoform (CD8αβ), as it can be seen in Fig. 3. The CD8α+/CD4+ ratio was high at 7 days postpartum,

TABLE I Number of T lymphocytes in NALT of 7-day-old rats

| | Markers | | | | | |
|---|---|---|---|---|---|---|
| | CD5+ | CD4+ | CD8α+ | CD8β+ | TCRαβ+ | TCRγδ+ |
| Mean ± SE* | 15.00 ± 0.97 | 7.17 ± 0.95 | 10.67 ± 0.99 | 8.83 ± 1.14 | 7.17 ± 0.65 | 10.67 ± 0.92 |

* $n = 6$. Two fields were recorded for each animal.

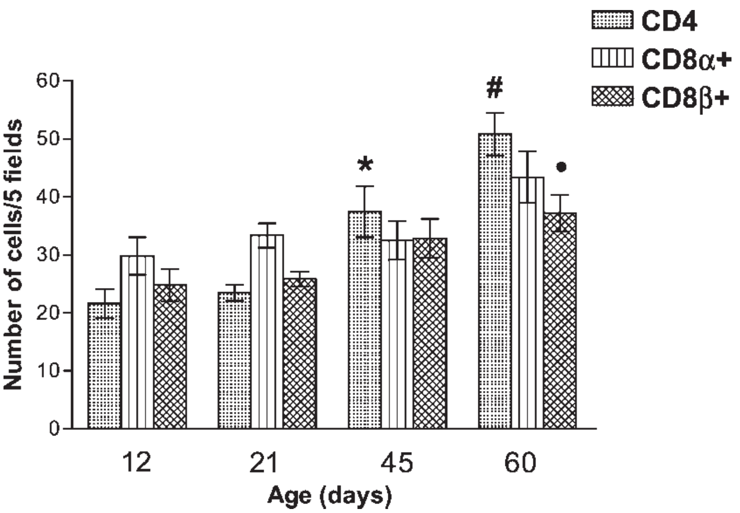

FIGURE 3 Number of CD4+, CD8α+ and CD8β+ T cells in NALT of 12-, 21-, 45- and 60-day-old rats. Bars represent mean ± SE of the number of cells in 5 fields from 5 to 6 animals. Tukey-Kramer: CD4: (*) 12, 21, 60 vs 45, $p < 0.05$; (#) 12, 21 vs 60, $p < 0.001$. CD8β: (•) 12, 21 vs 60, $p < 0.05$.

diminished with age and in 45-day-old rats reversed up to 60 days of age (Table II).

We found significant differences in TCRγδ only between 12 and 21 day-old rats (two-tailed Student's *t* test, $p = 0.0338$) and in the TCRαβ+ subset significant differences were observed from 12 to 60 days postpartum as shown in Fig. 4. γδ+ T cells outnumber those expressing TCRαβ from 7 to 45 days of age whereas in 45- and 60-day-old rats, αβ+ cells slightly outnumber γδ+ T cells. Consequently, at these ages the TCRγδ+/TCRαβ+ ratio reversed (Table II).

In order to ascertain the results obtained by immunohistochemistry, we performed a flow cytometric analysis of CD8α+ and TCRγδ+ subpopulations in 7- (C7) and 60-day old rats (C60). Both subsets were increased in the first group when compared to the latter (C7 vs C60): CD8α+: 21.0 vs 6.8%; TCRγδ+: 8.4 vs 0.95%; CD8α+ TCRγδ+: 7.5 vs 0.72%.

Lymphoid cells in NALT from 60 day-old rats are closely packed (Fig. 1C) and the overlying epithelium is infiltrated with intraepithelial lymphocytes. Many γδ+ lymphocytes in the lamina propria are shown in Fig. 1D.

### DISCUSSION

NALT is present at birth and some findings in mice concerning the mechanisms of organogenesis have recently been published (Harmsen *et al.*, 2002; Fukuyama *et al.*, 2002). There is only one report in mice regarding the postnatal development and structure up to 28 days of age (van der Ven and Sminia, 1993). Moreover, the early appearance of immunocompetent cells in Wistar rats has been described but only up to 21 days of age (Hameleers *et al.*, 1989). The latter is a semi-quantitative study indicating the existence, from day 4 to 10, of a predominance of the T helper subpopulation over the suppressor/cytotoxic one. An immunohistochemistry study in 15 week-old Wistar rats (Kuper *et al.*, 1990) and a cytometric analysis in 60 day-old Lewis rats have shown a predominance of T helper cells (Koornstra *et al.*, 1993). However, none of them has referred to TCRαβ and TCRγδ subpopulations during the postnatal development.

In the present study, the number of CD5+ T cells, as well as all its subpopulations, increases gradually with age, in parallel with the increase in size of this organ. A predominance of CD8α+ over CD4+ T cells was found from 7 to 45 days of age but from 45 to 60 days of age T helper cells outnumber the cytotoxic subpopulation. This finding is in agreement with the study performed in 60 day-old Lewis rats (Koornstra *et al.*, 1993). Similarly, NALT of adult chickens contains numerous CD4+ and scattered CD8+ T cells only in the large lymphoid accumulations present in the lamina propria under the respiratory epithelium (Ohshima and Hiramatsu, 2000).

Due to the lack of monoclonal antibodies there was no previous information on CD8 isoforms, as well as TCRαβ and TCRγδ. Therefore, the early appearance—from 7 to 60 days of age—of CD8α+ and CD8β+, TCRγδ+ and TCRαβ+ subpopulations was studied and is discussed in the present report.

In our study by immunohistochemistry, the most relevant finding is the predominance of γδ+ over αβ+ T cells from 7 to 45 days postpartum whereas in 45- and 60-day-old rats, αβ slightly outnumber γδ T cells. Moreover, in the present report cytometric studies have shown that the percentage of CD8α+ and γδ+ T cells, as

TABLE II Ratios between T-cell populations

| | Age (days) | | | | |
|---|---|---|---|---|---|
| Ratio | 7 | 12 | 21 | 45 | 60 |
| CD8α+/CD4+ | 1.49 | 1.38 | 1.42 | 0.87 | 0.85 |
| TCRγδ+/TCRαβ+ | 1.49 | 1.21 | 1.30 | 0.96 | 0.85 |

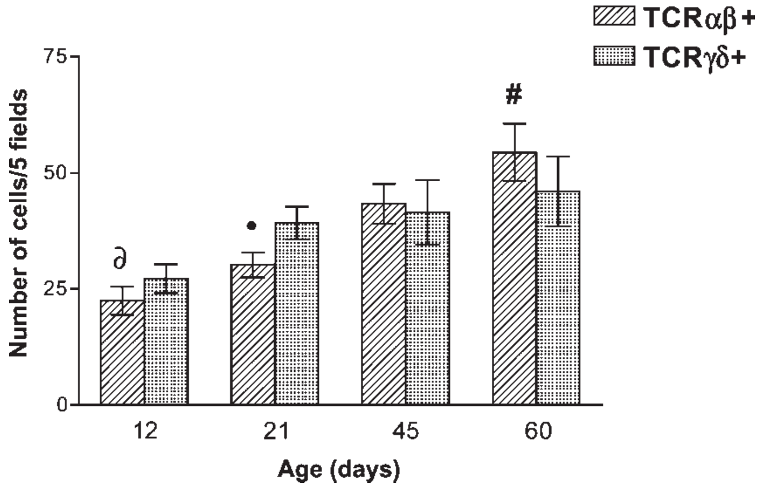

FIGURE 4 Number of TCRαβ+ and TCRγδ+ T cells in NALT of 12-, 21-, 45- and 60-day-old rats. Bars represent mean ± SE of the number of cells in 5 fields from 5 to 6 animals. Tukey-Kramer: TCRαβ: (∂) 12 vs 45, $p < 0.05$; (#) 12 vs 60, $p < 0.001$; (•) 21 vs 60, $p < 0.01$.

well as the population coexpressing both phenotypes (TCRγδ+CD8α+), are significantly higher at 7 days of age when compared to 60 day-old rats. Another relevant finding is that the majority of CD8+ T cells belong to the heterodimeric subset. This observation is in agreement with results obtained in mice (Heritage *et al.*, 1997).

Therefore, the present study shows that the pattern of development of T-cell subpopulations in NALT is similar to the one found previously in our laboratory when studying BALT (Márquez *et al.*, 2000).

It is well known that γδ T cells are involved in the immune surveillance and repair of damaged epithelia (Boismenu and Havran, 1994). Besides, it was suggested that γδ T cells provide immunoprotective functions in instances where αβ T cells do not, especially in young animals (Hayday, 2000). Therefore, the high number of γδ and CD8 T cells early in NALT development may indicate the importance of these subpopulations in the protection of the nasal mucosa in suckling and weaning Wistar rats.

### *Acknowledgements*

This paper is part of the Thesis of Gustavo Adolfo Sosa. The authors thank the Department of Nutrition for the use of animal facilities. This work was supported by grants PIP 4147 from CONICET, and TB72 and B064 from the University of Buenos Aires.

### *References*

Belai, A.A., El-Gohery, Y. and Talaat, M. (1977) “Nasal and paranasal pathology in experimental bilharziasis”, *J. Laryngol Otol.* **91**, 391–400.

Boismenu, R. and Havran, W.L. (1994) “Modulation of epithelial cell growth by intraepithelial gamma delta T cells”, *Science* **266**, 1253–1255.

Croitoru, K. and Bienenstock, J. (1994) “Characteristics and functions of mucosa-associated lymphoid tissue”, In: Ogra, P.L., Mestecky, J., Lamm, M.E., Strober, W., McGhee, J.R. and Bienenstock,, J., eds, Handbook of Mucosal Immunology (Academic Press, Orlando, USA), pp 141–149.

Diamond, G., Legarda, D. and Ryan, L.K. (2000) “The innate immune response of the respiratory epithelium”, *Immunol. Rev.* **73**, 27–38.

Fukuyama, S., Hiroi, T., Yokota, Y., *et al.* (2002) “Initiation of NALT organogenesis is independent of the IL-7R, LTβR, and NIK signalling pathways but requires the Id2 gene and $CD3^-CD4^+CD45^+$ cells”, *Immunity* **17**, 31–40.

Hameleers, D.M.H., van der Ende, M., Biewenga, J. and Sminia, T. (1989) “An immunohistochemical study on the postnatal development of rat nasal-associated lymphoid tissue (NALT)”, *Cell Tissue Res.* **256**, 431–438.

Harkema, J.R., Plopper, C.G., Hyde, D.M., Wilson, D.W., St George, J.A. and Wong, V.J. (1987) “Nonolfactory surface epithelium of the nasal cavity of the bonnet monkey: a morphologic and morphometric study of the transitional and respiratory epithelium”, *Am. J. Anat.* **180**, 266–279.

Harmsen, A., Kusser, K., Hartson, L., *et al.* (2002) “Organogenesis of nasal-associated lymphoid tissue (NALT) occurs independently of lymphotoxin-alpha (LT-α) and retinoic acid receptor-related orphan receptor-gamma, but the organization of NALT is LT-α dependent”, *J. Immunol.* **168**, 986–990.

Hayday, A.C. (2000) “γδ cells: a right time and a right place for a conserved third way of protection”, *Annu. Rev. Immunol.* **18**, 975–1026.

Heritage, P.L., Underdown, B.J., Arsenault, A.L., Snider, D.P. and McDermott, M.R. (1997) “Comparison of murine nasal-associated lymphoid tissue and Peyer’s patches”, *Am. J. Respir. Crit. Care Med.* **156**, 1256–1262.

Hodges, R.D. (1974) “The respiratory system. 1. The nasal cavity”, In: Hodges, R.D., ed., The Histology of the Fowl (Academic Press, London, England) **1**, pp 113–125.

Ichimiya, I., Kawauchi, H., Fujiyoshi, H., Tanaka, T. and Mogi, G. (1991) “Distribution of immunocompetent cells in normal nasal mucosa: comparisons among germ-free, specific pathogen-free, and conventional mice”, *Ann. Otol. Rhinol. Laryngol.* **100**, 638–642.

Kelemen, G. (1947) “The junction of the nasal cavity and the pharyngeal tube in the rat”, *Arch. Otolaryngol.* **45**, 159–168.

Koornstra, P.J., Duijvestijn, A.M., Vlek, L.F.M., Marres, E.H.M.A. and van Breda Vriesman, P.J.C. (1993) “Immunohistology of nasopharyngeal (Waldeyer’s ring equivalent) lymphoid tissue in the rat”, *Acta. Otolaryngol., (Stockh)* **113**, 660–667.

Kuper, C.F., Hameleers, D.M.H., Bruijntjes, J.P., van der Ven, I., Biewenga, J. and Sminia, T. (1990) “Lymphoid and non-lymphoid cells in nasal-associated lymphoid tissue (NALT) in the rat”, *Cell Tissue Res.* **259**, 371–377.

Loo, S.K. and Chin, K.N. (1974) “Lymphoid tissue in the nasal mucosa of primates, with particular reference to intraepithelial lymphocytes”, *J. Anat.* **117**, 249–259.

McDermott, M.R. and Bienenstock, J. (1979) “Evidence for a common mucosal immunologic system. I. Migration of B immunoblasts into intestinal, respiratory and genital tissues”, *J. Immunol.* **122**, 1892–1897.

Mair, T.S., Batten, E.H., Stokes, C.R. and Bourne, F.J. (1987) “The histological features of the immune system of the equine respiratory tract”, *J. Comp. Pathol.* **97**, 575–586.

Mair, T.S., Batten, E.H., Stokes, C.R. and Bourne, F.J. (1988) “The distribution of mucosal lymphoid nodules in the equine respiratory tract”, *J. Comp. Pathol.* **99**, 159–168.

Márquez, M.G., Sosa, G.A. and Roux, M.E. (2000) “Developmental study of immunocompetent cells in the bronchus-associated lymphoid tissue (BALT) from Wistar rats”, *Dev. Comp. Immunol.* **24**, 683–689.

Mazanec, M.B., Nedrud, J.G., Kaetzel, C.S. and Lamm, M.E. (1993) “A three-tiered view of the role of IgA in mucosal defense”, *Immunol. Today* **14**, 430–435.

Ohshima, K. and Hiramatsu, K. (2000) “Distribution of T-cell subsets and immunoglobulin-containing cells in nasal-associated lymphoid tissue (NALT) of chickens”, *Histol. Histopathol.* **15**, 713–720.

Pruitt, K.M., Rahemtulla, B., Rahemtulla, F. and Russell, M. (1999) “Innate humoral factors”, In: Ogra, P.L., Mestecky, J., Lamm, M.E., Strobe, W., Bienenstock, J. and McGhee, J.R., eds, Mucosal Immunology, 2nd Edn. (Academic Press, San Diego, USA), pp 65–88.

Sainte-Marie, G.A. (1962) “paraffin-embedding technique for studies employing immunofluorescence”, *J. Histochem. Cytochem.* **10**, 250–256.

Spit, B.J., Hendriksen, E.G.J., Bruijntjes, J.P. and Kuper, C.F. (1989) “Nasal lymphoid tissue in the rat”, *Cell Tissue Res.* **255**, 193–198.

Van der Ven, I. and Sminia, T. (1993) “The development and structure of mouse nasal-associated lymphoid tissue: an immuno- and enzyme-histochemical study”, *Reg. Immunol.* **5**, 69–75.

